# Efficacy and safety of novel oral anticoagulants in clinical practice: a report from three centers in Sweden

**DOI:** 10.1186/s12959-014-0029-6

**Published:** 2014-12-02

**Authors:** Ashkan Labaf, Martin Carlwe, Peter J Svensson

**Affiliations:** Department of Clinical Sciences, Lund University, Malmö, Sweden; Department of Cardiology, Skåne University Hospital, Malmö, Sweden; Department of Medicine, Hallands Hospital, Halmstad, Sweden; Department of Coagulation disorders, Skåne University Hospital, 205 02 Malmö, Sweden

**Keywords:** Novel oral anticoagulants, Dabigatran, Atrial fibrillation, Thromboembolism, Major bleeding

## Abstract

**Introduction:**

In clinical trials new oral anticoagulants (NOAC) have proved to be as effective as warfarin for thromboprophylaxis in atrial fibrillation. The aim of this study was to evaluate the efficacy and safety of these drugs in clinical practise.

**Methods and results:**

All patients treated with new oral anticoagulants at Skåne University hospital and Halland County Hospital Halmstad between 2009 and September 2013 was identified in the Swedish national quality registry for atrial fibrillation and anticoagulation (AuriculA). Medical records were reviewed to identify thromboembolism and major bleeding and compared to a warfarin cohort with a time in therapeutic range (TTR) of 76%. There were 826 patients, mean age 70.6, follow up 591 years, with atrial fibrillation treated with NOAC. Dabigatran was the initial drug in 79% of the cohort. The incidences of ischemic stroke/ TIA and major bleeding were 1.9 (95% CI; 1.0-3.2) and 2.0 (95% CI; 1.1-3.5) per 100 patient-years respectively. The corresponding incidences for warfarin were 1.5 (95% CI; 1.0-2.2) and 2.5 (95% CI; 1.8-3.3), with no statistical significance between the groups. Two subdural hematomas occurred 0.4 (95% CI; 0.06-1.1) per 100 patient-years. Mean age of patients with complications was 77.9 (±5.9) and 69.3 (±11.3) for those without (p < 0.001). The discontinuation rate was 6.5% and 1.7% was due to dyspepsia for dabigatran, lower than the RE-LY trial.

**Conclusion:**

This study, largely based on dabigatran shows that treatment is efficient and safe in everyday clinical practice and not significantly different compared to a warfarin cohort with tight anticoagulation control.

**Electronic supplementary material:**

The online version of this article (doi:10.1186/s12959-014-0029-6) contains supplementary material, which is available to authorized users.

## Background

Oral anticoagulation with warfarin is an effective treatment for prevention of ischemic stroke and systemic thromboembolism in atrial fibrillation (AF). However, the need of regular INR checks and significant interaction with diet and drugs are detrimental. These concerns have dramatically been modified with the introduction of the novel oral anticoagulants (NOACs) – dabigatran, a factor IIa (thrombin) inhibitor, and the factor Xa inhibitors rivaroxaban and apixaban [1-3]. The pharmacokinetics of these preparations is predictable, which means that frequent samplings similar to those that are necessary during treatment with warfarin are redundant. RE-LY (Randomized Evaluation of Long-Term Anticoagulant therapy) compared two doses of dabigatran etexilate against warfarin in AF patients with 1 or more stroke risk factors. The 150 mg b.i.d. was superior for the primary efficacy endpoint with similar a similar rate of bleeding whereas the 110 mg b.i.d. dose was noninferior to warfarin for the primary endpoint and superior to warfarin for major bleeding events. ROCKET AF (Rivaroxaban versus Warfarin in Non valvular atrial fibrillation) compared rivaroxaban once daily against warfarin and reported noninferiority for efficacy and safety.

The Danish registry study on dabigatran, showed that the results for dabigatran appear to persist even in everyday clinical practice [4]. However, this study used registers searching diagnoses according to The International Classification of Diseases (ICD) for extraction of data, including patient-related risk factors. All of the drugs have a degree of renal excretion, especially dabigatran which is why The European Society of Cardiology recommends that renal function should be monitored during treatment with NOAC [5]. Reduced glomerular filtration rate (GFR) with NOACs is associated with bleeding events [6].

The main purpose of this study was to evaluate the efficacy and safety of treatment with NOAC in three Swedish centers and compared with a historical cohort treated with warfarin. As a secondary objective, we assessed predictors of adverse events by a case control study.

## Material and methods

All patients treated with NOAC at Halland County Hospital Halmstad, Skåne University hospital in Lund and Malmö between 2009 and September 2013 was identified in the Swedish national quality registry for atrial fibrillation and anticoagulation (AuriculA). AuriculA was introduced in 2008 at our centres and now contains over 110,000 patients with more than 200 affiliated centers managing their daily dosing activity of oral anticoagulants, mainly warfarin, through the system. The program is web-based and includes decision support for dosing of warfarin. This methodology ensures that all patients treated with NOAC at these centres are included, thus reducing potential selection bias. All medical records and AuriculA were reviewed to obtain data on sex, indication for anticoagulation treatment, treatment time, complications and mortality. This cohort was compared to a historical cohort of patients from the same centres with warfarin from 2008, also by means of AuriculA [7]. The primary outcome were ischemic stroke, transient ischemic attack (TIA), systemic embolism, acute myocardial infarction (AMI) and major bleeding. AMI was defined as type 1 MI [8]. The International Society on thrombosis and Haemostasis definition on major bleeding was used (fatal bleeding, and/or symptomatic bleeding in a critical area of organ, such as intracranial or pericardial or intramuscular with compartment syndrome, and/or bleeding causes a fall in haemoglobin level of 20 g/L or more, or leading to transfusion of two or more units of red cells [9].

A case control study was performed to identify risk factors including kidney function, CHADS_2_, CHA_2_DS_2_-VASc and HAS-BLED for ischaemic stroke/TIA and major bleeding among patients with atrial fibrillation treated with a NOAC. The case group was defined as those who suffered of stroke/TIA or major bleeding event. The control group was defined as patients with NOAC treatment without an adverse event and was matched by age, sex and type of medication. For every case, three subjects were randomly selected as controls. Eleven cases with stroke/TIA were matched with 33 controls, and 12 cases with major bleeding were matched with 36 controls.

From the medical records, data on plasma creatinine, CHA2DS2-VASc and HAS-BLED were obtained for the case control group. Estimated-GFR (eGFR) was calculated by the Lund-Malmö equation [10]. The protocol was based on renal monitoring at treatment initiation, after 3, 6 and 12 months of treatment and thereafter annually except in subjects with reduced renal function who were monitored biannually.

### Statistical analysis

Categorical data were described by percentages and compared using the chi-square test, and Student’s t-test was used for continuous data. The days at risk were extracted from AuriculA for each patient and Incidence rates were calculated using the Person time module in Openepi, version 3.01 (www.openepi.com) with 95% confidence intervals. Multivariate logistic regression analysis was used to identify risk factors associated with thrombosis and major bleeding in the case control study. The control group were matched by age and gender and type of medication (Dabigatran and Rivaroxaban). All tests were performed two-tailed, and a p-value <0.05 was considered significant. All other analyses were performed using SPSS Statistics (Version 21; SPSS Inc., IBM Corporation, NY, USA).

## Results

In total 901 patients was identified in AuriculA. When medical records were reviewed it appeared that 11 had not taken the medication and were excluded. Another four were excluded due to unclear indication. Of the remaining 886 patients, 826 had the indication atrial fibrillation, and 58 had venous thromboembolism. Nine patients had both AF and venous thromboembolism (VTE) and are included in the AF group. Baseline characteristics and outcomes for all NOACS with AF are presented in Additional file 1: Table S1. Total time in treatment was 590 years of which 84 (10%) had a history of stroke or TIA. The most used drug was dabigatran, which was the initial drug in 78.5% of the patients. The dose was adjusted or altered to another NOAC in 5.8% of the patients. Patient that received dabigatran 110 mg were older than the 150 mg group, 77.2 (±8.5) vs 66.0 (9.2), p < 0.001 and the warfarin group, 75.0 (±9.6), p =0.002. The small group and few adverse events in the rivaroxaban groups were too small for analysis and conclusions. Apixaban was approved and introduced later and only a few patients were treated during this period.

The incidence of major bleeding and stroke/TIA events for the NOACs were not significantly different from the warfarin cohort. Twelve cases of major bleeding occurred. Seven gastrointestinal, 2 subdural haematomas, 1 muscular haematoma, 1 scalp wound and 1 critical bleeding in an eye. Another 2 occurred during surgery and invasive procedure and was excluded since it was iatrogenic and it was unclear whether the medication was discontinued as recommended, before the intervention or not. The incidence of gastrointestinal bleeding for all the NOACs combined was 1.2 (95% CI; 0.5-2.3) per 100 patient-years.

There were 6 ischemic strokes, 5 TIA and 2 AMI in the cohort. There was also one occlusion of an arteriovenous fistula in a patient treated with plasmapheresis. The latter was excluded because the lack of indication for NOAC-treatment. Mean age for patients with TE was 79.9 (±6.3) compared to those without TE 70.6 (±10.3), p =0.01. Corresponding ages for major bleeding was 77.2 (±5.6) and 70.6 (10.3), p =0.02 respectively.

Twelve deaths occurred during the treatment corresponding to an incidence of 2.0 (95% CI; 1.1-3.5) per 100 treatment years. Two were due to ischemic strokes, 3 in the final stage of malignancy, 3 cases of end-stage heart failure, 1 pneumonia, 1 gastroenteritis, and 1 due to an AMI.

Adverse events and discontinuation of the different drugs are presented in Additional file 1: Table S2. Adverse events not related to primary outcome was 5.9% and 4.4% for dabigatran 110 mg and 150 mg respectively. The most common adverse event was dyspepsia which occurred in 2.7% and 1.2% respectively, and lower than the RE-LY trial (11.8% and 11.3%). The discontinuation of dabigatran 110 and 150 mg were 7.3% and 6.1% respectively.

The case control group consisted of 22 subjects with 23 complications which is presented in Additional file 1: Table S3. Diabetes was significantly associated to major bleeding with the unadjusted odds ratio of 4.1 (95% CI; 1.0-16.8). CHADS_2_ and HAS-BLED was significantly higher in the cases with stroke/TIA compared to the controls and CHA_2_DS_2_-VASc had a borderline significant trend. Renal function was not significantly different between the cases and controls in neither group.

## Discussion

This present study, which includes all patients with NOAC-treatment in three major centres in Sweden, demonstrates the incidence of thrombotic, major bleeding and mortality in a clinical setting. We show that stroke/TIA and major bleeding were not significantly different between NOAC-treated patients and the warfarin-treated subjects with a TTR of 75.8%. In the RE-LY and ROCKET AF trials, mean TTR were 64% and 55%. The total incidence rate of all NOACs, which primarily consist of dabigatran and to a lesser extent rivaroxaban, for stroke/TIA and major bleeding, were 1.9 and 2.0 per 100 patient-years respectively. In the RE-LY trial, the rate of stroke/systemic embolism were 1.53%/year in the group that received 110 mg b.i.d. and 1.11%/year in the group that received 150 mg b.i.d. The bleeding events were 2.71%/year and 3.11%/year respectively. For comparison, the present study reported a higher incidence for the group that received dabigatran 110 mg b.i.d., stroke/TIA (3.8%/year) and major bleeding (3.3%/year). Patient that received dabigatran 110 mg were older than the 150 mg group, the warfarin group and older than the dabigatran 110 mg group in the RE-LY trial, (71.4 (±8.6), p < 0.001). The older age and associated comorbidities that accompanies might account for the increased rate of adverse events. In a subgroup analysis of the RE-LY [11], there was a highly significant interaction between treatment and age for major bleeding, such that patient older than 75 had less benefit on major bleeding than younger patients, irrespective of the renal function.

Compared with the warfarin group and RE-LY trial, the dabigatran 150 mg group was not significantly different. The incidence of stroke 1.0%/year were similar to the RE-LY (1.11%/year) and the warfarin group (1.5%/year). The bleeding rate of 1.6%/year was lower than the RE-LY trial (3.11%/year) but not significant, p = 0.16, and lower than the warfarin group (2.5%/year), p = 0.46. These data corresponds well with a subgroup analysis of the RE-LY [12], which demonstrated that 150 mg dabigatran was not superior at reducing the risk of stroke at higher TTR setting, as in our warfarin cohort. Further, the use of continuous aspirin in 40% of the patients in the RE-LY trial might partly have led to the higher bleeding rates. Aspirin was used in only two patients in the present study. Also, the RE-LY trial did not stratify the different doses during randomization according to the patient characteristics which may explain the differences in adverse events between our patients and RE-LY. This implies for instance that patients older than 75 and creatinine clearance between 30–45 ml/minute in RE-LY were treated with dabigatran 150 mg b.i.d.. The few intracranial bleeding (two subdural haematomas) that occurred (0.4%/year) is persistent with the randomized controlled studies concerning NOAC.

The adverse events were lower compared to the RE-LY trial. Dyspepsia which was the most common adverse event, occurred in 1.7% of the dabigatran patients compared to over 11% in the RE-LY trial. The observation and reporting of adverse effects are usually more accurate and careful in randomized controlled trials than in clinical practise which may explain some of the difference. Under-reporting of adverse drug reactions in real life is extensive and estimated to be in excess of 90% [13]. Nevertheless, the rate of adverse events seems not to outweigh the data from the RE-LY trial. The discontinuation rate of dabigatran was 6.5% (250 median treatment days) and due to primary outcome including death but also due to worsening renal failure and ablation treatment compared to the RE-LY trial which was 15% and 16% for each doses at one year of follow up. The patient’s decision for discontinuing was over 7% in RE-LY which could have been attributable to the increasing vigilance of the patients for a new study drug. The lower discontinuation rate in real life is however reassuring.

In the Danish “Real-World” study [4], the crude rates of stroke and major bleeding for dabigatran 150 mg were 3.5 and 2.2 per 100 patient-years respectively, and corresponding incidences for dabigatran 110 mg were 2.7 and 2.8 per 100 treatment years. The mean CHADS_2_ score was 1.2 and mean age 67.4 (dabigatran 150 mg) and 74.7 (dabigatran). A minor proportion of these patients could have represented AF cardioversions and ablations which reflect the real world clinical practise. However, an untold numbers of patients did not presumably satisfy the requirements for anticoagulation treatment for the prevention of stroke/systemic embolism. This would explain the higher adverse events for dabigatran 110 mg in our cohort, but not the lower incidence of stroke for dabigatran 150 mg group, (p = 0.02). Another recent single centre experience of dabigatran in Asia [14], showed surprisingly low incidence of adverse events, with only 2 major bleeding events (0.4%) and 1 stroke (0.2%). Recently FDA (The U.S. Food and Drug Administration) announced the new study in Medicare comparing dabigatran to warfarin which included 134,000 patients [15]. Dabigatran was associated with a lower risk of ischemic strokes, intracranial haemorrhage and death compared to warfarin. The MI risk was similar and an increased risk of major gastrointestinal bleeding were found in the patients with dabigatran treatment.

The renal function in the present study was strictly monitored in all patients according to the guidelines. This has not been presented in other real-life follow-up studies including the aforementioned studies. In fact, another Danish study showed that when recommendations set by the European Medicine Agency were not fulfilled, the incidence of adverse events were higher than warfarin-treated patients [16]. At our centres, all physicians that initiate treatment with any anticoagulant, will automatically notify the anticoagulation clinic. This course entails that specific protocols regarding renal function, adverse events, concomitant drugs are monitored and altered when needed.

The renal function was not significantly different between the cases and controls, probably as a result of selecting patients without renal impairment according to the guidelines, but also because of an active monitoring with adjustment of dose or discontinuation of the drug in patients with progressive renal failure. This also shows that surveillance of renal function is useful and works well at our units.

## Conclusions

The present study which is largely based on treatment with dabigatran, shows that these agents are effective in preventing stroke and systemic embolism in atrial fibrillation in a real life clinical setting, and not statistically different compared to a warfarin cohort with a high TTR (76%). With regular monitoring of renal function and thereafter adjusted dose, the complication rate is low and intracranial bleeding is rare. The adverse events and discontinuation rate was lower for dabigatran than in the RE-LY trial.

## Additional file

Additional file 1: Table S1. Primary endpoints according to the different novel oral anticoagulants and comparison to the warfarin cohort. **Table S2.** Adverse events not related to primary outcome and discontinuation of the different drugs and doses. **Table S3.** CHADS_2_, CHA_2_DS_2_-VASc, HAS-BLED and eGFR in the case control study.
