# Supplementary material for: Efficacy and safety of novel oral anticoagulants in clinical practice: a report from three centers in Sweden
Source: Thromb J. 2014 Dec 2;12:29. doi: 10.1186/s12959-014-0029-6 (PMC4265344; doi:10.1186/s12959-014-0029-6)
Supplement: Additional file 1: Table S1. — Primary endpoints according to the different novel oral anticoagulants and comparison to the warfarin cohort. Table S2. Adverse events not related to primary outcome and discontinuation of the different drugs and doses. Table S3. CHADS2, CHA2DS2-VASc, HAS-BLED and eGFR in the case control study. [file 12959_2014_29_MOESM1_ESM.docx]

|  | Dabigatran | Dabigatran | Rivaroxaban | Rivaroxaban | Apixaban | Apixaban | Total NOACs | Warfarin | P-value |
| --- | --- | --- | --- | --- | --- | --- | --- | --- | --- |
|  | 110 mg | 150 mg | 15 mg | 20 mg | 2.5 mg | 5 mg |  |  |  |
| N | 218 | 432 | 39 | 133 | 1 | 3 | 826 | 2002 |  |
| Age (SD) | 77.2 (8.5) | 66.0 (9.2) | 81.9 (7.3) | 71.1 (8.1) | - | - | 70.5 (10.3) | 75.0 (9.6) |  |
| Sex (female) | 94 (43) | 153 (35) | 16 (41) | 47 (35) |  |  | 310 (38) | 854 (42.7) |  |
| Time in treatment (days) | 66,813 | 114,972 | 6567 | 26,064 | 88 | 667 | 215,171 |  |  |
| Years | 183 | 315 | 18 | 71 | 0.2 | 2 | 590 | 1766 |  |
| Stroke/TIA ^a^ | 7 | 3 | 1 |  |  |  | 11 | 26 |  |
| Event rate* | 3.8 (1.7–7.6) | 1.0 (0.2-2.6) | 5.6 (0.3-27) | - |  |  | 1.9 (1.0–3.2) | 1.5 (1.0–2.2) | 0.62 |
| Major Bleeding | 6 | 5 |  | 1 |  |  | 12 | 44 |  |
| Event rate* | 3.3 (1.3–6.8) | 1.6 (0.6-3.5) | - | 1.4 (0.07-7.0) |  |  | 2.0 (1.1–3.5) | 2.5 (1.8–3.3) | 0.65 |
| GI-bleeding ^b^ | 3 | 3 |  | 1 |  |  |  |  |  |
| Event rate* | 1.6 (0.4-4.5) | 1.0 (0.2-2.6) | - | 1.4 (0.07-7.0) |  |  |  |  |  |
| MI ^c^ | 1 |  |  | 1 |  |  |  |  |  |
| Event rate* | 0.5 (0.03-2.7) |  |  | 1.4 (0.07-7.0) |  |  |  |  |  |

Additional file 1

**Table S1** Primary endpoints according to the different novel oral anticoagulants and comparison to the warfarin cohort

* Incidence (95% CI); per 100 patient-years, ^a^ Transient ischemic attack, ^b^ Gastrointestinal bleeding, ^c^ Myocardial infarction.

**Table S2** Adverse events not related to primary outcome and discontinuation of the different drugs and doses

|  | Dabigatran 110 mg | Dabigatran 150 mg | Rivaroxaban 15 mg | Rivaroxaban 20 mg |
| --- | --- | --- | --- | --- |
| Adverse events | 5.9 (13) | 4.4 (19) | 10.3 (4) | 1.5 (2) |
| Dyspepsia | 2.7 (6) | 1.2 (5) |  |  |
| Allergy | 0.5 (1) | 1.2 (5) | 2.6 (1) | 0.7 (1) |
| Dyspnea | 0.5 (1) | 0.2 (1) |  |  |
| Muscle pain | 0.5 (1) | 0.2 (1) |  |  |
| Headache | 0.5 (1) | 0.2 (1) | 2.6 (1) |  |
| Diarrhea | 0.5 (1) |  |  |  |
| Vertigo |  |  | 2.6 (1) |  |
| Other | 0.5 (1) | 1.4 (6) |  |  |
| Minor bleeding | 0.5 (1) |  | 2.6 (1) |  |
| Discontinuation | 7.3 (16) | 6.1 (26) | 2.6 (1) | 3.7 (5) |
| Outcome | 2.7 (6) | 0.9 (4) | 2.6 (1) | 2.2 (3) |
| Renal failure | 2.3 (5) | 1.2 (5) |  |  |
| Ablation | 0.5 (1) | 2.3 (10) |  | 0.7 (1) |
| Other | 1.8 (4) | 1.6 (7) |  | 0.7 (1) |
| Changed dose | 0.5 (1) | 2.3 (10) |  | 0.7 (1) |

Values are presented as % (n).

**Table S3** CHADS_2_, CHA_2_DS_2_-VASc, HAS-BLED and eGFR in the case control study

| mean (SD) | Stroke/TIA (n = 12) | Matched controls (n = 33) | p-value | Major bleeding (n = 12) | Matched controls (n = 36) | p-value |
| --- | --- | --- | --- | --- | --- | --- |
| CHADS_2_ | 2.8 (±1.1) | 2.0 (±0.7) | 0.006 | 2.6 (±1.0) | 2.2 (1.2) | 0.34 |
| CHA_2_DS_2_-VASc | 4.2 (±1.6) | 3.4 (±1.0) | 0.075 | 3.75 (±1.42) | 3.6 (1.6) | 0.71 |
| HAS-BLED | 2.0 (±1.10) | 1.4 (±0.8) | 0.045 | 1.9 (±0.67) | 1.6 (0.8) | 0.127 |
| eGFR* | 62.0 (±15.7) | 59.1 (±12.8) | 0.54 | 55.2 (±17.4) | 58.0 (±10.8) | 0.50 |

*estimated glomerular filtration rate (ml/min/1.73 m^2^).
